# Novel ulcerative leg lesions in yearling lambs: Clinical features, microbiology and histopathology

**DOI:** 10.1016/j.vetmic.2020.108790

**Published:** 2020-08

**Authors:** G.J. Staton, H. Crosby-Durrani, G. Roberts, J.S. Duncan, N. Seechurn, R. Blowey, N.J. Evans

**Affiliations:** a Department of Infection Biology & Microbiomes, Institute of Infection, Veterinary & Ecological Sciences, University of Liverpool, Leahurst Campus, Chester High Road, Neston, CH64 7TE, United Kingdom; b George Farm Vets, High Street, Malmesbury, Wiltshire, SN16 9AU, United Kingdom; c Department of Livestock & One Health, Institute of Infection, Veterinary & Ecological Sciences, University of Liverpool, Leahurst Campus, Chester High Road, Neston, CH64 7TE, United Kingdom; d School of Veterinary Science, Institute of Infection, Veterinary & Ecological Sciences, University of Liverpool, Leahurst Campus, Chester High Road, Neston, CH64 7TE, United Kingdom; e Minsterworth, Gloucester, GL2 8JG, United Kingdom

**Keywords:** Sheep, Ulcerative dermatitis, *Fusobacterium necrophorum*, *Streptococcus dysgalactiae*

## Abstract

Here we report an outbreak of an atypical, ulcerative dermatitis in North Country mule lambs, located in South Gloucestershire, UK. The lesions, which appeared to be contagious, occured between the coronary band and the carpal joint as a focal, well demarcated, circular, ulcerative dermatitis. Histopathological examination of the lesion biopsies revealed areas of ulceration, epidermal hyperplasia, suppurative dermatitis and granulation tissue. Clumped keratohyalin granules and intracellular keratinocyte oedema (ballooning degeneration) were evident within lesion biopsies, consistent with an underlying viral aetiology. A PCR-based microbiological investigation failed to detect bovine digital dermatitis-associated treponeme phylogroups, *Dichelobacter nodosus*, *Staphylococcus aureus*, *Dermatophilus congolensis* or *Chordopoxvirinae* virus DNA. However, 3 of the 10 (30 %) and 6 of 10 (60 %) lesion samples were positive for *Fusobacterium necrophorum* and *Streptococcus dysgalactiae* DNA, respectively. Contralateral limb swabs were negative by all standard PCR assays. To better define the involvement of *F. necrophorum* in the aetiology of these lesions, a qPCR targeting the rpoB gene was employed and confirmed the presence of *F. necrophorum* DNA in both the control and lesions swab samples, although the mean *F. necrophorum* genome copy number detected in the lesion swab samples was ∼19-fold higher than detected in the contralateral control swab samples (245 versus 4752 genome copies/μl, respectively; P < 0.001). Although we have not been able to conclusively define an aetiological agent, the presence of both *F. necrophorum* and *S. dysgalactiae* in the majority of lesions assayed supports their role in the aetiopathogenesis of these lesions.

## Highlights

• An outbreak of an infectious dermatological disorder of unknown aetiology in a flock of yearling lambs was investigated.
• Lesions occurred on the distal limb between the coronary band and carpel joint as a circular ulcerative dermatitis.
• *Treponema* spp., *Dichelobacter nodosus*, *Staphylococcus aureus, Dermatophilus congolensis* and poxvirus screens were negative.
• *Fusobacterium necrophorum* and *Streptococcus dysgalactiae* were detected in the majority of lesions examined.
• An aetiology involving bacterial infection with *F. necrophorum* and *S. dysgalactiae* was implicated.

## 1 Introduction

Infectious dermatological disorders of unknown aetiology, for which treatment modalities have not been defined, are a significant challenge to the clinician and an animal welfare concern. In the UK, a series of reports have been made describing unusual skin lesions of sheep that exclusively affect the lower limbs and appear to be contagious. In all cases, the lesions grossly resemble strawberry footrot, yet the causative organisms, orf parapox virus and *Dematophilus congolensis*, have not been detected (APHA, 2019) and the aetiology of these lesions remains elusive. The first reported outbreak occurred in the Autumn of 2008 with a separate outbreak occurring in October 2010 (van der Burgt et al., 2011). In both outbreaks, the lesions responded sub-optimally to topical antibiotics and systemic tetracyclines, although systemic treatment with tilmicosin (Micotil, Elanco) and long-acting amoxicillin was successful. Similar outbreaks were again reported in March and July of 2019 (APHA, 2019). Here, we report a further outbreak of an atypical, infectious ulcerative dermatitis of the lower limbs in sheep. In terms of the gross pathological and epidemiological features, this outbreak is comparable to those previously described and affected 200 (8%) of 2500 yearling North Country mule lambs, located in South Gloucestershire, UK, in August 2019. Lesions typically presented on the lateral aspect of the lower limbs, between the coronet and the carpus/tarsus. These encrusted circular lesions were large, ulcerated and susceptible to bleeding. Affected animals were isolated and treatment with long-acting amoxicillin (Betamox™ LA, Norbrook) appeared to resolve the lesions, although a second dose of amoxicillin was occasionally required (Roberts, 2019; personal communication); a bacterial aetiology was therefore suspected. This appeared to be the third such outbreak on the same holding: in August 2017, an outbreak of ulcerative dermatitis in 200 North Country mule lambs sold to the farm was recorded, which was followed by a second, comparable outbreak in yearling lambs in August 2018. Employing bacteriological and histopathological techniques, we sought to examine the aetiological and pathological features of the most recent outbreak and alert clinicians to the recurrence of this apparently novel skin disorder of the lower limb.

## 2 Methods

All samples were collected in August 2019 as part of a diagnostic investigation into a dermatological lesion outbreak of unknown aetiology in yearling lambs. The lambs were living on a single farm in South Gloucestershire, UK. Ten affected animals were selected for sampling, from which both the lesion and a healthy, contralateral limb were sampled non-invasively using a sterile cotton swab firmly rotated onto the sampling site. In addition, punch biopsies were collected from four of the affected animals for diagnostic purposes and stored in 10 % formalin for histopathological examination.

### 2.1 Microbiological culture

Due to a suspicion of BDD-associated *Treponema* phylogroup involvement, lesion swabs were transported to the laboratory in Oral Treponeme Enrichment Broth (OTEB, Anaerobe Systems, Morgan Hill, CA, USA) for selective culture. Each swab head was inoculated into 7 mL of OTEB, supplemented with 10 % Fetal Calf Serum and antibiotics (rifampicin (5 μg/mL) and enrofloxacin (5 μg/mL)). Inoculations were incubated for >48 h at 37 °C under anaerobic conditions, until bacterial growth was evident. Cultures were examined by phase contrast microscopy and bacterial genomic DNA isolated from OTEB cultures as described previously (Clegg et al., 2015). To aid bacterial species identification, a PCR targeting the universal 16S rRNA gene was performed as described previously (Rurangirwa et al., 1999) and the amplicon submitted for commercial Sanger sequencing (Source Bioscience, Nottingham). Additionally, charcoal skin swabs and lesion scab material were submitted to the Animal and Plant Health Agency (APHA), Shrewsbury, UK, for bacterial culture and electron microscopy, respectively.

### 2.2 Diagnostic PCR assays

Bacterial genomic DNA was extracted from the lesion and control swabs using the QIAquick DNeasy™ blood and tissue kit (Qiagen, Manchester, UK), according to manufacturer’s instructions. All samples were subjected to the following PCR assays as described previously: *Treponema* genus (Moore et al., 2005), *Treponema medium* phylogroup (Evans et al., 2009a), *Treponema phagedenis* phylogroup (Evans et al., 2009a), *Treponema pedis* phylogroup (Evans et al., 2009a), *Fusobacterium necrophorum* (Bennett et al., 2009) *Dichelobacter nodosus* (Sullivan et al., 2015), *Staphylococcus aureus* (Graber et al., 2007), *Streptococcus dysgalactiae* (Phuektes et al., 2001), *Dermatophilus congolensis* (Shaibu et al., 2010), orf parapox virus (Tedla et al., 2018) and high-GC and low-GC content pan-chordopoxvirus PCRs (Li et al., 2010). All PCRs were performed on a Mastercycler Gradient thermocycler (Eppendorf, Germany). Each 20 μL PCR reaction contained 1 μL of DNA template and was performed using *Taq* polymerase (Solis Biodyne, Tartu, Estonia) according to manufacturer’s instructions. All PCRs were performed in duplicate with a relevant genomic DNA positive control (as described below) and a water negative control. Amplicons were visualised by gel electrophoresis and ethidium bromide staining. Statistical analysis was performed using the Fisher’s exact test in Minitab v. 18 (Minitab Inc., PA, USA).

### 2.3 PCR validations and controls

Control material used in the following diagnostic PCR assays were as described previously: *Treponema* genus and the BDD-associated treponeme assays (Evans et al., 2009a), *F. necrophorum* and *D. nodosus* (Sullivan et al., 2015). Genomic bacterial DNA, extracted using Chelex® 100 resin, was obtained from pure cultures of *S. aureus*, *S. dysgalactiae* and *D. congolensis,* supplied by the University of Liverpool Veterinary Clinical Microbiology Diagnostics service (Liverpool, UK), as described previously (Chua et al., 2005). Orf parapox virus DNA was extracted directly from the orf parapox vaccine (Scabivax Forte™, MSD Animal Health, Summit, NJ, USA) using QIAamp Viral RNA Mini Kit (Qiagen, Manchester, UK) for use with the orf parapox PCR and high-GC content pan-chordopoxvirus PCR. Finally, cowpox DNA was extracted from a feline skin lesion using the DNeasy Blood and Tissue Kit (Qiagen, Manchester, UK) for use with the low-GC content pan-chordopoxvirus PCR. DNA concentration and purity were determined using the Nanodrop™ 2000 spectrophotometer (Thermo Scientific, Hemel Hempstead, UK). PCR specificity was determined empirically for the *S. aureus*, *S. dysgalactiae*, *D. congolensis*, Orf parapox and pan-chordopoxvirus diagnostic PCR assays using a gradient thermocycler (Eppendorf, Germany) to test a range of annealing temperatures. Amplicons generated from clinical sample PCRs were submitted for Sanger sequencing (Source Bioscience, Nottingham).

### 2.4 16S rRNA gene Sanger sequencing

Three lesion swab DNA extractions were additionally subjected to 16S rRNA gene PCR and the resultant amplicons were submitted for Sanger sequencing in an attempt to identify the most abundant bacterial species within these sample, as described previously (Evans et al., 2009b). Amplicons were sequenced commercially (Source Bioscience, Nottingham, UK). DNABaser v. 5.15 (Heracle BioSoft SRL, Romania) was employed for sequence assembly. Contigs were submitted to BLASTn (Altschul et al., 1990) for species identification.

### 2.5 qPCR assays

The *F. necrophorum* bacterial load (genome copy number) was estimated by qPCR as described previously using an assay that targets an 86 bp (base pair) sequence within the RNA polymerase beta subunit (rpoB) gene of both subspecies; F. *necrophorum* subsp. *necrophorum* and subsp. *funduliforme* (Witcomb et al., 2014). In addition, a qPCR targeting a 61 bp sequence within the RNA polymerase sigma subunit (rpoD) gene of *D. nodosus* was performed as described previously (Calvo-Bado et al., 2011). Primer and probe sets were synthesised and purified commercially (TIB MOLBIOL, GmbH, Berlin, Germany) and serial dilutions of *D. nodosus* and *F. necrophorum* DNA were prepared to provide an estimated 6310,000–6,310 and 43,700–437 genome copies/μl, respectively. Standards and clinical samples were analysed in triplicate on a 7900 HT FAST Real Time PCR system (Applied Biosystems). Samples that failed to generate fluorescence after 40 qPCR cycles were classified as negative. Statistical analysis was performed using the Mann Whitney *U* test in Graphpad Prism v. 5.03 (San Diego, USA).

### 2.6 Histopathology

Punch biopsies were collected from the lesion edge of four affected sheep under local anaesthetic (Adrenacaine™, Norbrook) and preserved in 10 % neutral buffered formalin. After fixation, the tissues were trimmed to a thickness of 3−5 mm, processed routinely and embedded in paraffin wax. Sections (5 μM) were stained with haematoxylin and eosin (HE), Gram and Warthin Starry stains, using standard methods, to aid the identification of relevant bacterial colonisation.

## 3 Results

**Fig. 1:**
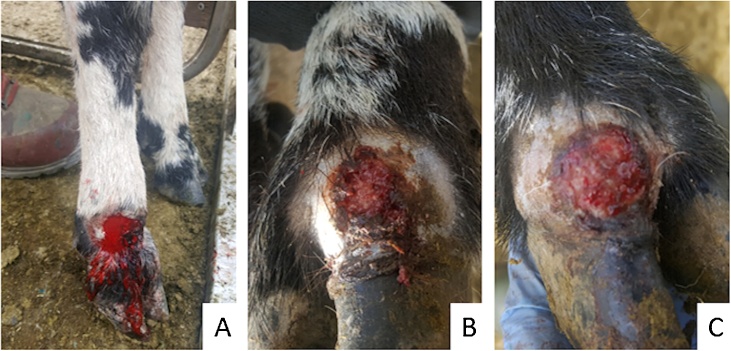
Macroscopic description of lesions. Lesions typically presented on the lateral aspect of the lower limbs, between the coronet and the carpus/tarsus, as a focal, well demarcated, ulcerative dermatitis with haemorrhage and occasional scab or central granulation tissue formation. Lesions from three affected sheep are shown (A–C).

Lesions were similar in gross appearance (Fig. 1). All affected the skin on the distal limb between the coronary band and the carpal joint as a focal, well demarcated, round (up to 4 cm in diameter), ulcerative dermatitis with haemorrhage and sometimes with scab or central granulation tissue formation. Occasionally the lesion appeared near to the coronary band.

### 3.1 Microbiological culture

After 48 h, there was no evidence of spirochaetal growth in selective medium. Heavy growth of coccobacilli was noted in all cultures. The resultant cultures were subjected to 16S rRNA gene PCR and sequencing which yielded 1412 bases of unambiguous consensus sequence. A BLASTn search revealed an alignment, with 96.18 % sequence identity, to *Enterococcus faecium* strain AHC4 16S ribosomal RNA gene. Using selective medium, the APHA was able to culture *S. dysgalactiae* from three of the six charcoal swabs submitted for analysis. Electron microscopic analysis of lesion scab material failed to detect virions.

### 3.2 Diagnostic PCR assays

The results of the PCR-mediated analyses are shown in Table 1. The dataset shows the presence (+) or absence (−) of specific PCR products as determined by relevant diagnostic assays.

**Table 1:** Results of the diagnostic PCR assays.

Sheep eartag no.	Sample type	*Treponema* genus	BDD *Treponema* Phylogroup^a^			*F. necrophorum*	*D. nodosus*	*S. aureus*	*S. dysgalactiae*	*D. congolensis*	Orf parapox virus	Pan-chordopoxvirus
			1	2	3							
2325	Healthy limb	–	–	–	–	–	–	–	–	–	–	–
07486	Healthy limb	–	–	–	–	–	–	–	–	–	–	–
08055	Healthy limb	–	–	–	–	–	–	–	–	–	–	–
08215	Healthy limb	–	–	–	–	–	–	–	–	–	–	–
08140	Healthy limb	–	–	–	–	–	–	–	–	–	–	–
05929	Healthy limb	–	–	–	–	–	–	–	–	–	–	–
08210	Healthy limb	–	–	–	–	–	–	–	–	–	–	–
08070	Healthy limb	–	–	–	–	–	–	–	–	–	–	–
1840	Healthy limb	–	–	–	–	–	–	–	–	–	–	–
02376	Healthy limb	–	–	–	–	–	–	–	–	–	–	–
2325	Lesion	–	–	–	–	–	–	–	+	–	–	–
07486	Lesion	–	–	–	–	–	–	–	–	–	–	–
08055	Lesion	–	–	–	–	+	–	–	+	–	–	–
08215	Lesion	–	–	–	–	–	–	–	+	–	–	–
01840	Lesion	–	–	–	–	+	–	–	–	–	–	–
05929	Lesion	–	–	–	–	–	–	–	–	–	–	–
08210	Lesion	–	–	–	–	–	–	–	+	–	–	–
08070	Lesion	–	–	–	–	–	–	–	+	–	–	–
08410	Lesion	–	–	–	–	+	–	–	–	–	–	–
02376	Lesion	–	–	–	–	–	–	–	+	–	–	–

−, not detected; +, detected.

a BDD *Treponema* phylogroup: Group 1, *T.* medium phylogroup; Group 2, *T. phagedenis* phylogroup; Group 3, *T. pedis phylogroup*.

### 3.3 Amplicon Sanger sequencing

To confirm the specificity of the *S. dysgalactiae* PCR, clinical sample amplicons were submitted for commercial Sanger sequencing. Contig assembly generated 251-bp of unambiguous consensus sequence which aligned with 98.86–99.29 % sequence identity to the 16 S–23 S ribosomal RNA intergenic spacer region of *S. dysgalactiae* subsp. *dysgalactiae* (SDSD) strain NCTC 10238 using BLASTn (Altschul et al., 1990). A lesser sequence identity for *S. dysgalactiae* subsp. *equisimilis* (SDSE) was generated (98.58–98.61 %), indicating that SDSD was the likely subspecies of *S. dysgalactiae* in these lesion samples.

The results of the diagnostic PCR assays are shown in Table 1. All lesion swab samples were negative for BDD-associated treponeme phylogroups, *D. nodosus*, *S. aureus*, *D. congolensis* and orf parapox virus and pan-chordopoxvirus diagnostic PCR assays. Conversely, lesion sample analysis by the *F. necrophorum* and the *S. dysgalactiae* diagnostic PCR yielded positive results in 3 of 10 (30 %; P > 0.05) and 6 of 10 (60 %; P < 0.05) lesion samples, respectively. No target DNA was detected in control swabs by any assay.

### 3.4 16S rRNA gene Sanger sequencing

Three lesion sample swab DNA extracts were subjected to eubacterial 16S rRNA PCR and the resultant amplicons were submitted for commercial Sanger sequencing (Source Bioscience, Nottingham, UK). Contig assembly generated 820 and 934 bases of high quality, unambiguous sequence for sample 08055 and sample 08070, respectively; sample 08215 failed to generate high-quality sequencing reads. Pairwise sequence analysis using BLASTn generated alignments with 100 % and 99.79 % sequence identity to *F. necrophorum* subs. *funduliforme* strain F1260 for samples 08055 and sample 08070, respectively. Because alignments were also successfully generated for *F. necrophorum* subspecies *necrophorum* (100 % sequence identity for 08055 and 99.79 % identity for 08070), subspecies differentiation was not possible.

### 3.5 qPCR

In agreement with the findings of the diagnostic *D. nodosus* PCR, all samples were negative for *D. nodosus* by qPCR. Conversely, *F. necrophorum* was detected in 9 of 10 (90 %) of the contralateral limb control swab samples and 7 of 10 (70 %) of the lesion swab samples, although the mean copy number in the lesion samples was 19-fold greater than that of the contralateral control swab samples (245 versus 4752 genome copies/μl, respectively; P < 0.001).

### 3.6 Histopathological examination

The results of a histopathological examination of the lesions is summarised in Table 2. All lesions had areas of ulceration, epidermal hyperplasia, suppurative dermatitis and granulation tissue. Three quarters of the samples had responses that suggested chronic irritation as demonstrated by the clumped keratohyalin granules. These samples also had intracellular oedema (ballooning degeneration) of the keratinocytes that is usually suggestive of viral involvement, although no further evidence of a viral aetiology was generated by the microbiological techniques employed in this investigation.

**Table 2:** Histological examination of lesion biopsy material revealed a number of pathological changes associated with this presentation. A suppurative dermatitis associated with ulceration, epidermal hyperplasia and granulation tissue were seen in all cases. Other histopathological changes were seen to varying degrees.

Sheep eartag no.	08070	08210	05929	08055
Ulceration	+	+	+	+
Vesicle	–	–	–	+
Epidermal hyperplasia	+	+	+	+
Hyperkeratosis	–	+	–	–
Clumped keratohyline granules	+	+	–	+
Keratinocyte intracellular oedema	+	+	–	+
Intracytoplasmic protein	–	+	–	+
Spongiosis	+	+	–	+
Granulation tissue	+	+	+	+
Suppurative dermatitis	+	+	+	+

−, not observed; +, observed.

**Fig. 2:**
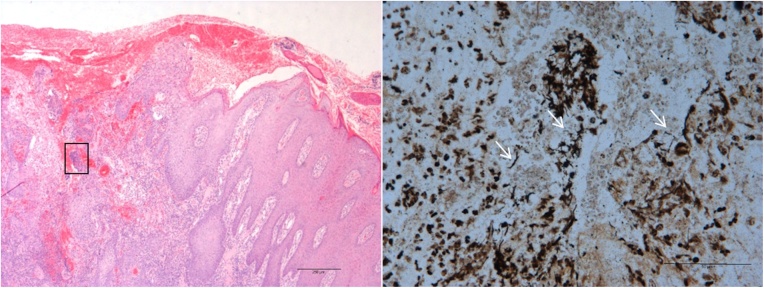
Histopathological examination of a lesion punch biopsy section using Hematoxylin and eosin (H&E) and Warthin Starry (WS) staining. H&E stain of a lesion punch biopsy section (A). The black-boxed area, stained with WS, has been magnified to high power (B) to reveal bacterial cells of varying morphology (arrows).

The results of the Haematoxylin and eosin and Warthin Starry staining are shown in Fig. 2 and demonstrated small colonies of mixed bacterial morphology mostly 1 × 2−3 μm coccobacilli to rods, often in short chains. Bacteria were mostly associated with the site of ulceration.

## 4 Discussion

As part of this study into the aetiology of the ulcerative dermatitis outbreak in sheep, the lesions were screened for a range of pathogenic microorganisms relevant to dermatological disorders of the ovine lower limb using PCR-based diagnostic assays. The results generated suggest that *D. congolensis*, *S. aureus*, the BDD-associated treponemes, orf parapox virus and *D. nodosus* are unlikely to be involved in lesion pathogenesis. Our findings closely resemble those reported following a detailed investigation by the APHA into the aetiology of comparable outbreaks in 2019, which failed to find evidence of *Treponema* (commensal or pathogenic), *D. nodosus* or *D. congolensis* colonisation in lesion material (APHA, 2019). Furthermore, no poxvirus was identified by PCR and no virus was detected by electron microscopy, and a diagnosis of strawberry footrot (potentially caused by these organisms) has thus been excluded. However, our investigation identified the presence of two bacterial species of interest in the lesion swab material, namely *F. necrophorum* and *S. dysgalactiae*, which may contribute towards the aetiopathogenesis of these atypical lesions or at least modify the expression of an underlying (and as yet, unknown) disorder.

An association between these lesions and *S. dysgalactiae* was initially suspected following the successful culture and isolation of this organism from 3 (50 %) of the charcoal lesion swabs submitted to the APHA for analysis, and later confirmed by our own PCR-based assay. *S. dysgalactiae* was also cultured from lesion samples collected from the March 2019 outbreak (APHA, 2019), although not the outbreaks reported by van der Burgt et al. (2011). In addition, an association between these lesion swabs and *F. necrophorum* was demonstrated by PCR-based methods and eubacterial 16S rRNA gene sequencing. We were also able to culture *E. faecium* from lesion scrapings, although its relevance is unclear. *E. faecium* has been demonstrated to be one of the most abundant facultative anaerobes in ruminants (Lauková, 1994) and, given its commensal association with the gastrointestinal tract and its ubiquity in the farm environment via faecal contamination, a role in the instigation of these lesions is considered improbable.

The role played by *S. dysgalactiae* in this outbreak, although associated with 60 % of the lesions, is uncertain. No dermatological disorder of sheep resembling the presentation described here, in which *S. dysgalactiae* is considered to be an aetiological agent, exists. These lesions may therefore represent a new presentation, or their colonisation with *S. dysgalactiae* may be secondary or incidental. There exists a paucity of data relating to the characteristics and pathogenesis of *S. dysgalactiae*. In 1996, *S. dysgalactiae* was divided into two subspecies: *S. dysgalactiae* subspecies *equisimilis* (SDSE) and *S. dysgalactiae* subspecies *dysgalactiae* (SDSD). SDSE are large colony β-haemolytic streptococci belonging to Lancefield Group C or Group G and associated predominantly with human *S. dysgalactiae* infections whereas SDSD are α-haemolytic streptococci belonging to Lancefield Group C or L and are almost exclusively animal-restricted pathogens. *S. dysgalactiae* has been frequently associated with bovine mastitis (Aarestrup and Jensen, 1996), and is the most common cause of infectious polyarthritis (joint ill) in lambs aged up to four weeks (Watkins and Sharp, 1998). Cases of arthritis, meningitis and endocarditis in piglets (Woods and Ross, 1977) have also been attributed to *S. dysgalactiae*, as has neonatal mortality in puppies (Vela et al., 2006). Thus, *S. dysgalactiae* infections are characterised by a broad host range and a diverse clinical spectrum. However, studies investigating the pathogenic capability and tissue tropism of SDSD are still limited and have focused predominantly on infections of the mammary epithelium. In relation to infections of the skin, SDSD may require specific predisposing factors (for example, microabrasions) to enable successful invasion. Recently, it was shown that bovine SDSD isolates were capable of human keratinocyte attachment and infection *in vitro* (Roma-Rodrigues et al., 2016), suggesting that the ovine epidermis may also be a target for infection.

Significantly, *S. dysgalactiae* exhibits the epidemiological characteristics of a transmissible pathogen, for instance, in cases of contagious bovine mastitis, where it may spread from cow to cow via fomites during milking (Fox and Gay, 1993). In addition, S. dysgalactiae carriage within the the vaginal tract of healthy ewes has also been reported (Rutherford et al., 2014), indicating that this bacterium may occupy a commensal niche. The primary source of *S. dysgalactiae* in this ulcerative dermatitis outbreak may therefore have been the lambs themselves, presumably from colonised sites distal to the lower limb. The reason why only 60 % of the lesions tested were positive for *S. dysgalactiae* are unclear. This result may reflect the true microbiological profile, in which *S. dysgalactiae* is only present in a subset of cases or PCR sensitivity may have been inadequate. Further work is required to understand precisely the relationship between *S. dysgalactiae* and this presentation.

*F. necrophorum* is a Gram-negative, non-spore-forming rod-shaped and pleomorphic anaerobe and a commensal inhabitant of the rumen (Nagaraja et al., 2005). *F. necrophorum* is classified as the primary pathogen of a number of diseases, including calf diphtheria (Panciera et al., 1989), hepatic abscesses (Lechtenberg et al., 1988) and interdigital phelgmon (Berg and Loan, 1975). Footrot in sheep, although considered caused by primary infection with *D. nodosus*, is exacerbated by *F. necrophorum* infection (Witcomb et al., 2014). Similarly, it has been speculated that *F. necrophorum* also contributes to the enhanced pathology of ‘non-healing’ bovine foot lesions (Staton et al., 2020). Thus, similarly to SDSD, *F. necrophorum* may also behave as an opportunistic, secondary coloniser. Interestingly, in bovine mastitis, *S. dysgalactiae* has been identified as being one of the first species of bacteria to colonise the bovine teat, which then favours secondary infection with other anaerobes, including *F. necrophorum* (Jonsson et al., 1991), a finding of potential relevance to the disease under investigation. In total, three methods were used to screen these lesions for *F. necrophorum*. Originally, a PCR targeting the leukotoxin (IktA) gene revealed that three (30 %) of the lesion swabs were positive for *F. necrophorum* DNA. However, genotypic identification by 16S rRNA gene sequencing of total PCR products revealed that two of the three lesion swabs analysed by this method contained *F. necrophorum* DNA, suggesting that this is the most abundant microorganism within these samples. A qPCR method, presumed to be of greater sensitivity than the conventional PCR, was therefore employed. This analysis detected *F. necrophorum* DNA in 9 of 10 (90 %) of the control swabs and 7 of 10 (70 %) of the lesion swabs, although the mean *F. necrophorum* genome copy number in the control swabs was ∼19-fold lower than in the lesion swab samples (245 versus 4752 genome copies/μl, respectively), indicating that *F. necrophorum* colonisation of intact skin was limited. The low-level detection of *F. necrophorum* in the control limb swab samples likely represents cross-contamination of disease-free sites from the exudative lesions on infected limbs. The limited association between the lesions and *F. necrophorum* as determined by IktA PCR potentially reflects a suboptimal level of sensitivity relative to the qPCR or genetic differences in the *F. necrophorum* bacterial populations between samples, in which some strains may be IktA-negative. Similarly to *S. dysgalactiae*, *F. necrophorum* has been delineated into two pathogenic subspecies; subsp. *necrophorum* and subsp. *funduliforme*, with the latter considered less pathogenic and involved in mixed infections (Lechtenberg et al., 1988). We were unable to discriminate subspecies using either the IktA or the RpoB assays. IktA/leukotoxin is considered to be the major virulence factor involved in invasive fusobacterial infections and is cytotoxic to bovine and ovine neutrophils, macrophages and hepatocytes (Tan et al., 1994). However, carriage of the IktA gene may be less common in non-bovine invasive animal strains (Ludlam et al., 2009), which may account for the disparity in detection frequency observed in this study.

Histopathological examination showed mostly non-specific features such as ulceration and chronic repair as demonstrated by the formation of granulation tissue and the clumpy keratohyalin granules. The observation of the intracellular oedema (ballooning degeneration) of keratinocytes was interesting. This finding is usually associated with diseases of a viral aetiology. However multiple microbiological techniques were unable to confirm viral involvement and special stains performed on the histopathological samples only confirmed the presence of mixed bacterial colonies.

Although we have shed additional light on the microbiology of these unusual lesions in yearling lambs, further studies are required to elucidate the precise aetiopathogenesis of this infection. Moreover, the apparent summer seasonality of disease expression identified in this case warrants further investigation. A compromised skin barrier, via umbilical severance, castration, tail docking or ear-tagging have all been suggested as potential risk factors for joint ill, and by implication, *S. dysgalactiae* infection, whilst thistles have been associated with orf parapox transmission because of their ability to abrade the skin (Watson, 2004). APHA, in 2019, similarly proposed that a traumatic aetiology precipitated by stemmy rye grass may account for the outbreak of unusual skin lesions. It’s noteworthy that in the case described here, some thistles were present in the permanent set-stocked pasture, although there was no history of any foreign body damage and no sharp edges were identified on fences or handling equipment.

In summary, we report an outbreak of an unusual ulcerative dermatitis in yearling lambs which closely resembles three other outbreaks in the United Kingdom. Although we have not been able to precisely define an aetiological agent, the presence of both *F. necrophorum* and *S. dysgalactiae* in a majority of the lesions assayed supports their role in the aetiopathogenesis of these lesions. Particular attention should be paid to both *F. necrophorum* and *S. dysgalactiae*, either of which may be a primary invader or part of a sequential infection involving a presently unknown primary pathogen. Further studies are required to delineate the role of these anaerobic bacteria in the pathogenesis of this disorder.
